# Composites Based on Electrodeposited WO_3_ and TiO_2_ Nanoparticles for Photoelectrochemical Water Splitting

**DOI:** 10.3390/ma17194914

**Published:** 2024-10-08

**Authors:** Ramunas Levinas, Elizabeth Podlaha, Natalia Tsyntsaru, Henrikas Cesiulis

**Affiliations:** 1 Faculty of Chemistry and Geosciences, Vilnius University, Naugarduko Str. 24, LT-03225 Vilnius, Lithuania; natalia.tintaru@chf.vu.lt (N.T.); henrikas.cesiulis@chf.vu.lt (H.C.); 2 State Research Institute, Center for Physical Sciences and Technology (FTMC), Saulėtekio Ave. 3, LT-10257 Vilnius, Lithuania; 3 Department of Chemical and Biomolecular Engineering, Clarkson University, 8 Clarkson Ave., Potsdam, NY 13699, USA; 4 Institute of Applied Physics, Moldova State University, MD-2028 Chisinau, Moldova

**Keywords:** tungsten trioxide titania composite, electrodeposition, photoelectrochemical water splitting, IMPS of OER

## Abstract

Photoelectrochemically active WO_3_ films were fabricated by electrodeposition from an acidic (pH 2), hydrogen-peroxide-containing electrolyte at −0.5 V vs. SCE. WO_3_-TiO_2_ composites were then synthesized under the same conditions, but with 0.2 g/L of anatase TiO_2_ nanoparticles (⌀ 36 nm), mechanically suspended in the solution by stirring. After synthesis, the films were annealed at 400 °C. Structural characterization by XRD showed that the WO_3_ films exhibit the crystalline structure of a non-stoichiometric hydrate, whereas, in WO_3_-TiO_2_, the WO_3_ phase was monoclinic. The oxidation of tungsten, as revealed by XPS, was W^6+^ for both materials. Ti was found to exist mainly as Ti^4+^ in the composite, with a weak Ti^3+^ signal. The efficiency of the WO_3_ films and composites as an oxygen evolution reaction (OER) photo-electrocatalyst was examined. The composite would generate approximately three times larger steady-state photocurrents at 1.2 V vs. SCE in a neutral 0.5 M Na_2_SO_4_ electrolyte compared to WO_3_ alone. The surface recombination of photogenerated electron–hole pairs was characterized by intensity-modulated photocurrent spectroscopy (IMPS). Photogenerated charge transfer efficiencies were calculated from the spectra, and at 1.2 V vs. SCE, were 86.6% for WO_3_ and 62% for WO_3_-TiO_2_. Therefore, the composite films suffered from relatively more surface recombination but generated larger photocurrents, which resulted in overall improved photoactivity.

## 1. Introduction

The adverse environmental impact of using fossil fuels for energy needs and generating H_2_ for chemical processing has led to significant research toward finding alternative and sustainable energy carriers, such as hydrogen (H_2_), with H_2_ produced by solar water splitting is more advantageous compared to other forms of green hydrogen [1,2,3]. The photoelectrochemical (PEC) water-splitting process includes light harvesting, photogenerated charge separation, and surface catalysis, and is an effective strategy as an alternative to fossil fuels. The reduction of water to produce hydrogen is coupled with the more kinetically hindered oxygen evolution reaction, which is typically carried out on metal oxide photoanodes [4,5]. Although TiO_2_ is recognized as an effective inexpensive photocatalyst in the UV range, [6,7], the addition of tungsten trioxide (WO_3_) is widely known to lower the band gap (~2.75 eV) and hence, allows absorption to occur within the visible range of the solar spectrum, [8,9,10,11]. However, WO_3_ alone suffers from high recombination of photon-generated electron–hole pairs [12] and is susceptible to photocorrosion caused by peroxo-species formed on the surface during the photoelectrochemical reaction [13]. Tungsten trioxide doped by TiO_2_ shows a red-shifted optical response in the visible light range due to a band gap decrease from a lowering of the conduction band edge, and can act as a photocatalyst. Momeni and Nazari [14] reported a change in band gap from 3.23 eV for bare TiO_2_ to 2.78 eV for WO_3_-doped TiO_2_ nanotubes. Also, doping TiO_2_ with cations like tungsten (W^6+^) introduces energy levels in TiO_2_ just below the conduction band that acts as an electron trap increasing the lifetime of charge carriers, and thus retarding recombination [15].

Heterojunctions of WO_3_/TiO_2_ layers have been used to increase the efficiency of the photocatalysts [16,17,18,19,20] by aligning the band structures of the two coupled semiconductors to encourage migration of photogenerated electrons (e^−^) and holes (h^+^) in separate directions across the heterojunction boundary, which reduces the electron–hole recombination. The highly oxidative valence band of WO_3_, arising from the O 2p orbitals, also helps to drive oxidation reactions, such as PEC oxygen evolution or degradation of dyes. WO_3_/TiO_2_ composites have been reported to perform as stable and efficient photoanodes in water splitting [21,22,23]. Numerous reports of dye degradation with tungsten doped TiO_2_ or heterojunctions are reported through the reactions of a photogenerated superoxide [24,25,26,27,28]. One such heterojunction unique to electrodeposition is the work presented by Martin et al. [29]. They electrodeposited WO_3_ onto a metallic Ti electrode then heat treated it to create a WO_3_/TiO_2_/Ti layered material that exhibited high photocurrent values toward the photoelectrocatalytic degradation of propyl paraben. An alternative strategy to reduce recombination rates is to apply a potential or bias and photogenerated electrons are drawn away from the electrode–electrolyte interface through the external surface. This electrically enhanced photocatalysis was observed for electrochemically prepared TiO_2_/WO_3_ layers on stainless steel for the oxidation of organics [30].

Nanostructuring is also important for generating quantum confinement effects that alter the electron and hole transport behaviors, shifting of the electronic band structure, and the large surface-to-volume ratio can significantly increase the surface reaction sites [31]. However, it can also introduce defects or additional grain boundaries that can promote recombination [32].

As mentioned, synergistic effects, as a result of combining different photoactive materials, provide a promising pathway to improve the photoelectrochemical performance in heterojunction devices for solar water splitting [33]. To this end, a nanocomposite WO_3_ -TiO_2_ film was electrodeposited with a WO_3_ matrix incorporating nanoparticles of TiO_2_, in contrast to layered materials or those with a matrix of TiO_2_. The electrodeposition of WO_3_ films followed the approach by Pauporté [34], where the electrochemical deposition mechanism is multistaged and occurs through the reduction of a peroxy-tungstate species as outlined in Equations (1) to (3) [35,36,37].

(1) $${2WO}_{4}^{2-}+{4H}_{2}O_{2}\to W_{2}O_{11}^{2-}+{2OH}^{-}+{3H}_{2}O$$

(2) $$W_{2}O_{11}^{2-}+\left(2+3x\right)H^{+}+3xe^{-}\to 2H_{x}WO_{3}+\frac{\left(2+x\right)}{2}H_{2}O+\frac{\left(8-x\right)}{4}O_{2}$$

(3) $$H_{x}WO_{3}\to {WO}_{3}+xH^{+}+xe^{-}$$

In this study, the approach is modified by adding TiO_2_ nanoparticles to the sodium tungstate–hydrogen peroxide electrolyte. The electrodeposition is then carried out to simultaneously electrodeposit a WO_3_ film and capture TiO_2_ nanoparticles, resulting in a one-step synthesis of a composite with improved photoactivity for water splitting. Moreover, the use of an advanced non-stationary photoelectrochemical technique (IMPS) is demonstrated to probe charge carrier photogeneration in the space-charge layer and to elucidate important kinetic constants for charge transfer and recombination.

## 2. Materials and Methods

### 2.1. Composite Electrodeposition

**Figure 1:**
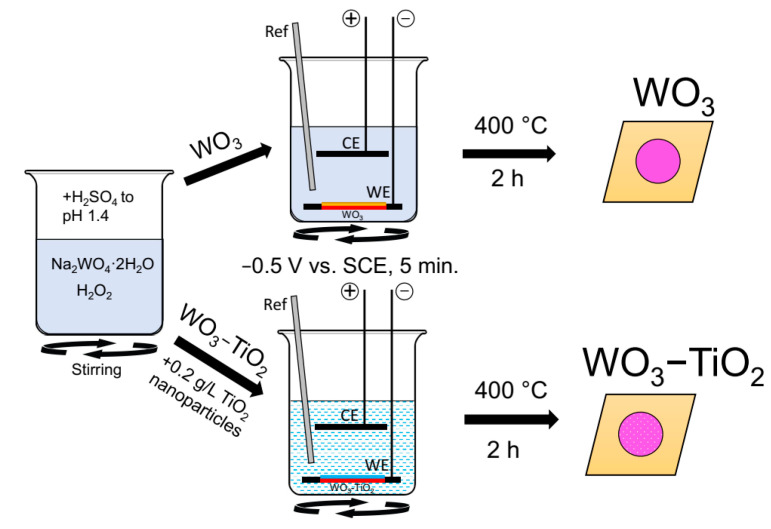
Schematic representation of material synthesis and annealing process.

A schematic representation of the cell and process used to deposit WO_3_ and WO_3_-TiO_2_ films is shown in Figure 1. All used chemicals were of ACS reagent grade. The electrolyte to electrodeposit WO_3_ films was prepared from 0.025 M Na_2_WO_4_·2H_2_O (Fisher Chemical, Hampton, NH, USA), 0.075% (*v*/*v*) H_2_O_2_ (Fisher Chemical, Hampton, NH, USA) which was 1.25 mL of 30% hydrogen peroxide, and the pH was adjusted to 1.4 with sulfuric acid (Fisher Cchemical, Hampton, NH, USA). To electrodeposit composite WO_3_-TiO_2_ films, the same electrolyte composition was used, but additionally, a particle loading of 0.2 g/L of anatase TiO_2_ (Thermo Fisher Scientific, Waltham, MA, USA) of average ⌀ 36 nm was added. The nanoparticles were mechanically suspended in the electrolyte by using a magnetic stir bar. Electrodeposition was carried out in a three-electrode cell, with a stainless-steel (type 316) working electrode (plates of 2 cm × 2 cm dimensions were used as the substrate, with a circular working area of 1.226 cm^2^), a Pt counter electrode, and an SCE reference electrode. The substrate was positioned in a holder parallel to the bottom of the electrodeposition bath. Deposition occurred during an applied potential of −0.5 V vs. SCE for 5 min.

### 2.2. Structural and Surface Morphology Characterization

The as-deposited WO_3_ and WO_3_-TiO_2_ films were thermally annealed at 400 °C for two hours, with a 2 °C min^−1^ temperature ramp-up rate. The surface morphology was characterized by a Hitachi TM 3000 scanning electron microscope (Hitachi Ltd., Tokyo, Japan). XRD diffraction patterns were obtained with a Rigaku MiniFlex II x-ray diffractometer (Rigaku Corporation, Tokyo, Japan).

The XPS analyses were carried out with a Kratos Axis Supra spectrometer (Kratos Analytical Limited, Manchester, UK), using a monochromatic Al K(alpha) source (15 mA, 15 kV). Survey scan analyses were carried out on the area of 300 × 700 µm at a pass energy of 160 eV. High-resolution analyses were also carried out on the area of 300 × 700 µm, but at a pass energy of 20 eV. The XPS signal due to adventitious carbon located at 284.8 eV was used as a binding energy (BE) reference.

### 2.3. PEC Characterization

The photoelectrochemical response of the resulting deposits was examined with linear sweep voltammetry, chronoamperometry, and IMPS, using a ZAHNER Zennium CIMPS-QE/IPCE system (Zahner-Elektrik, Kronach, Germany). The photoelectrochemical cell included a quartz window for front-illumination of the films, a platinum counter electrode, and an SCE reference electrode. A neutral 0.5 M Na_2_SO_4_ electrolyte was used. The light source was a variable-intensity LED, emitting monochromatic 365 nm UV light.

A typical characterization procedure of an electrochemically deposited and annealed WO_3_ or WO_3_-TiO_2_ film consisted of these steps:

Voltammetry experiments were conducted from 0.2 V to 1.6 V (vs. SCE) at 5 mV s^−1^, with a 4-s on/off illumination pulse (2 s on/2 s off). Chronoamperometry at a constant potential of 1.2 V used an illumination pulse of 120 s (60 s on/60 s off). Light intensity was set to 30 mW cm^−2^. IMPS was measured at steady state potentials: 0.6 V, 0.8 V, 1.0 V, 1.2 V (100 mW cm^−2^ light intensity, 10% amplitude modulation, 10 kHz to 0.1 Hz).

## 3. Results and Discussion

### 3.1. Structure and Surface Morphology

**Figure 2:**
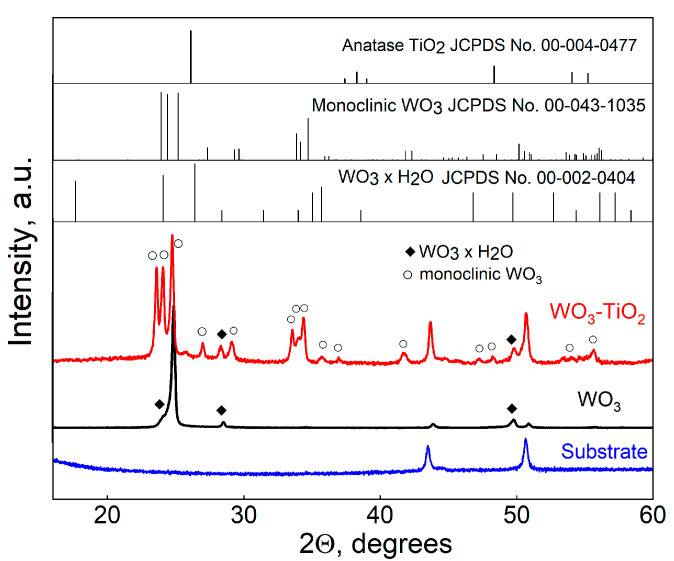
XRD patterns of stainless steel substrate and electrodeposited WO_3_ and WO_3_-TiO_2_ composite films, after annealing at 400 °C.

Figure 2 shows the XRD patterns of electrodeposited WO_3_ and WO_3_-TiO_2_ films that had been annealed at 400 °C for 2 h. The XRD pattern of the annealed stainless steel substrate is also included. It contains two peaks characteristic of austenite iron (JCPDS No. 00-023-0298) at 43.5° and 50.7°, and the substrate peaks also emerge in the diffractograms of the deposited films. The diffractograms of WO_3_ and WO_3_-TiO_2_ reveal the radically different phase structure of the films. WO_3_-TiO_2_ displays all the characteristic peaks of monoclinic WO_3_ [38,39]. However, there are a couple of relatively low-intensity peaks that cannot be attributed to this phase, in particular, at 27.6° and 49.5°. The analysis of WO_3_ reveals the origin of these peaks. The diffractogram of WO_3_ contains a very intense peak at ~ 24.1°, which may correspond to the (200) face of monoclinic WO_3_. There is also a broad hump at a slightly lower 2Θ of 23.3°, and two small peaks at 27.8° and 49.5°. These reasonably correspond to the unidentified peaks from WO_3_-TiO_2_ and have been attributed to hydrated forms of WO_3_ in the literature [40,41]. It is claimed that the formation of WO_3_·xH_2_O can occur through tuning of the intermediate peroxo-complex and thus is related to the H_2_O_2_ content in the solution [40]. The electrodeposition of a hydrated tungsten oxide has also recently been reported under conditions very similar to those used in this study [42]. Therefore, the formation of monoclinic WO_3_ when electrodepositing the composite must have been related to an equilibrium shift in the intermediate peroxo-complex caused by the interaction of solution species with nanoparticulate TiO_2_, possibly solvation. The crystallite sizes of WO_3_, as approximated by the Scherer equation, are 41 ± 9 nm for WO_3_ and 27.4 ± 3.3 nm for WO_3_-TiO_2_. Regarding the presence of TiO_2_, in the diffractogram of the composite film, a very faint hump at ~25° can perhaps be distinguished, which could be related to the main (101) peak of anatase TiO_2_.

**Figure 3:**
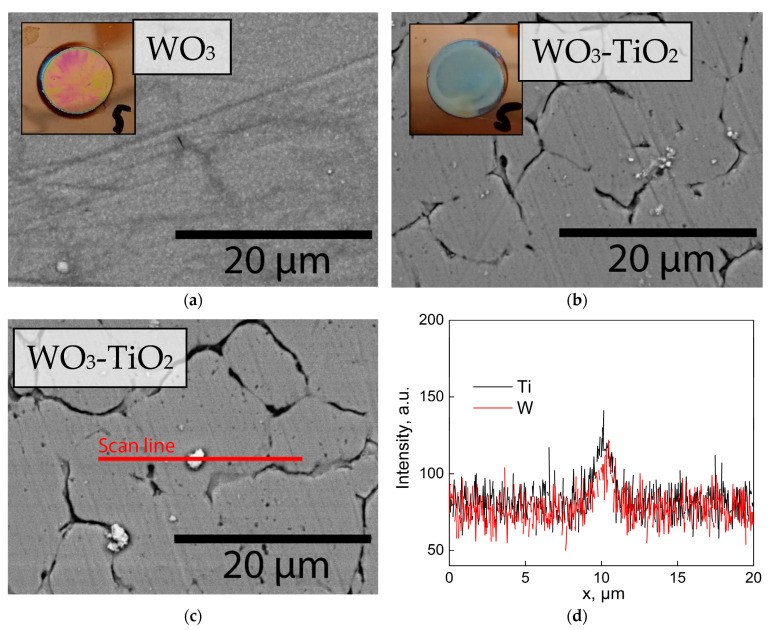
SEM surface morphology images of (**a**) WO_3_; (**b**) WO_3_-TiO_2_ films electrodeposited for 5 min, (**c**) SEM image of EDX line scan on a WO_3_-TiO_2_ film, (**d**) EDX line scan data portraying titanium and tungsten signals.

The visual appearance and SEM surface morphology images of WO_3_ and WO_3_-TiO_2_ films are shown in Figure 3. The WO_3_ film (Figure 3a) is inhomogeneously polychromic, which is indicative of a compact thin film of slightly varying thicknesses. The surface morphology, as observed by SEM, is smooth and fine-grained, as was also observed by Pauporté when first reporting on WO_3_ electrodeposition by this method [34]. For both films, signs of emerging cracks can be seen. It was noted earlier [43,44] that cracking and delamination occur if the layer is thicker (i.e., after longer deposition times). Accordingly, the WO_3_-TiO_2_ film is already cracked (Figure 3b), which may indicate a larger thickness. Scattered agglomerates of white particles, which are most likely TiO_2_, can be observed on the surface. Visually, the film appears “cloudy” because TiO_2_ particles had settled at the surface of the film during deposition. These particles were not always well-adhered to the surface. If the electrodeposition time was prolonged, they would detach from the surface when immersed in an electrolyte for further characterization. For this reason, the PEC characterization was carried out on films that had been deposited for 5 min.

The results of an EDX line scan (Figure 3c,d) show that a TiO_2_ nanoparticle agglomeration was observed on the surface. Titanium was detected, albeit with a very weak signal. It is also interesting to note that the tungsten signal increases along with titanium. This may simply be a topographical effect (i.e., a stronger signal obtained from a point closer to the detector), but it could also indicate that WO_3_ had electrodeposited onto TiO_2_.

**Figure 4:**
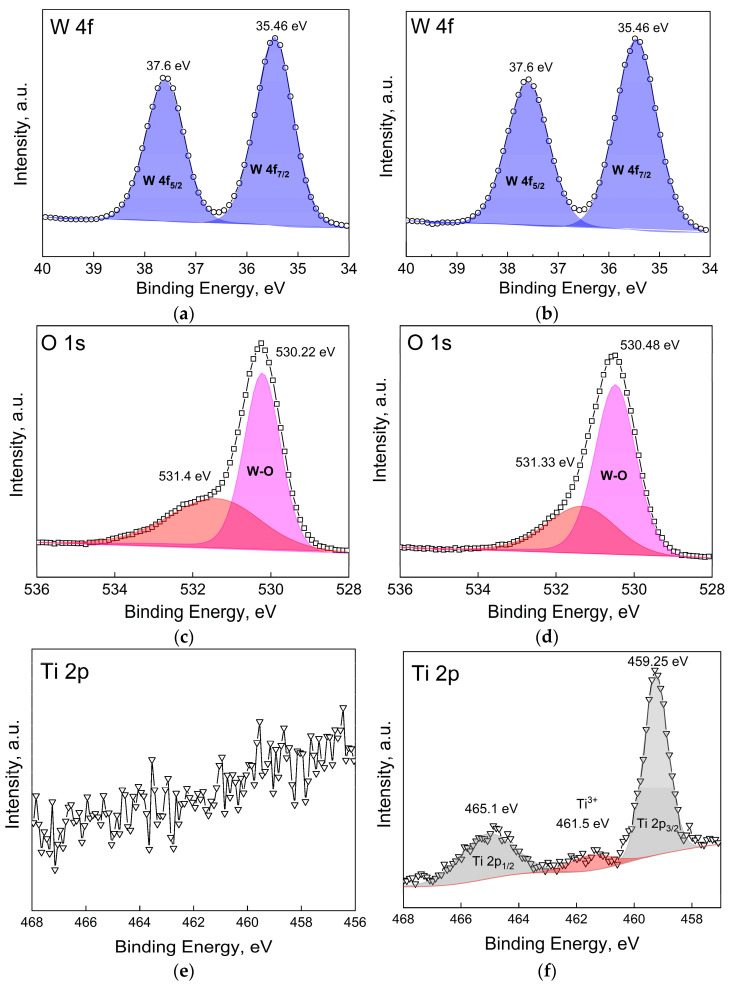
Deconvoluted core-level XPS spectra of W 4f, O 1s, and Ti 2p of a WO_3_ film (**a**,**c**,**e**) and WO_3_-TiO_2_ film (**b**,**d**,**f**).

The near-surface electronic structure of the WO_3_ and WO_3_-TiO_2_ films was investigated by XPS. It was found that for both films, the core-level structure of W 4f deconvolutes into a single doublet with W 4f_7/2_ and W 4f_5/2_ peaks at binding energies of 35.46 eV and 37.6 eV, respectively (Figure 4a,b). The position of these peaks, and the spin-orbit splitting energy of 2.14 eV, indicate the total dominance of the W^6+^ oxidation state [45,46]. Interestingly, the O 1s core-level spectra reveal that oxygen can be found in at least two oxidation states (Figure 4c,d). The O 1s peak seen here at 530.22 eV for WO_3_ and 530.48 eV for WO_3_-TiO_2_ is commonly attributed to lattice oxygen (i.e., the W-O bond) [47].

The origin of the second much broader peak at ~531.4 eV is slightly more complicated to explain. First and foremost, this cannot be Ti-related O because (1) the lattice oxygen peak energy is expected to be around 529–530 eV [48] and (2) the same peak also exists for WO_3_ without TiO_2_. A couple of other possibilities that are often reported are oxygen from non-stoichiometric WO_3-x_ or -OH groups incorporated within the lattice, which can result in peaks in the O 1s spectra at approximately 532.5 eV [47,49]. However, these energies also do not match. A very recent work by T. J. Frankcombe and Y. Liu describes what is most likely the cause of these peaks [50]. They found that a peak at this energy (commonly ascribed in the literature to oxygen vacancies) is instead caused by O 1s electrons from water molecules that are chemisorbed to the surface or surface oxygen passivated by hydrogen. This agrees well with the W 4f spectra, which show 100% dominance of W^6+^ and no trace of reduced oxidation states that would be expected with a large number of oxygen vacancies. The existence of chemisorbed water may also be related to the observation of hydrates in XRD characterization (Figure 2). Lastly, no Ti is found on WO_3_, and only a very weak Ti 2p signal is obtained from the WO_3_-TiO_2_ film (Figure 4e,f). The core level spectrum deconvolutes into one doublet with peaks at 459.25 eV and 465.1 eV, corresponding to Ti^4+^ from anatase TiO_2_ [51]. An indistinct hump at 461.5 eV may also be observed. It is on the verge of being noise, but a peak at this position has been attributed to Ti^3+^ [52]. The reduction of titanium dioxide nanoparticles is feasible under cathodic conditions in an acidic electrolyte, so the signal is attributed to Ti^3+^ here as well.

### 3.2. Photoelectrochemical Characterization

**Figure 5:**
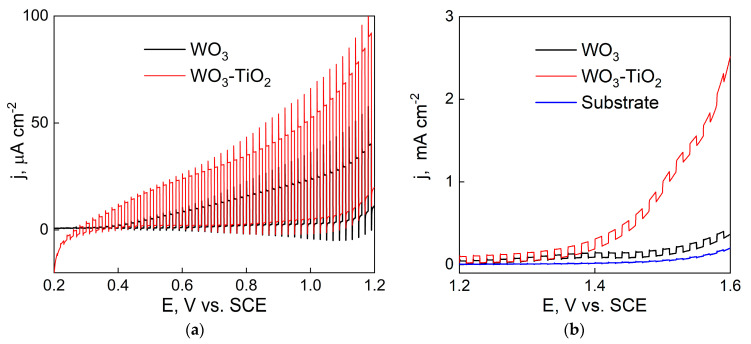
Chopped UV LSV measurements at low potentials (**a**) and high potentials (**b**) in 0.5 M Na_2_SO_4_ of WO_3_ and WO_3_-TiO_2_ films deposited within 5 min; 5 mV s^−1^, 30 mW cm^−2^ light intensity.

Initially, the PEC properties of WO_3_ and WO_3_-TiO_2_ films were characterized by carrying out a potential scan with intermittent on/off light pulses (Figure 5a). Two differences are immediately apparent: (1) the WO_3_-TiO_2_ film generates larger photocurrents throughout the entire potential range and (2) the photocurrent onset potential of the composite is more cathodic (~ 0.2 V vs. SCE) in comparison to just WO_3_ (~ 0.35 V vs. SCE). As expected, the magnitude of the photocurrent increases with the applied potential as the space-charge layer, where the photoexcitation of charge carriers occurs and becomes wider due to the increasing strength of the applied electrical field. It is also worth noting that both WO_3_ and WO_3_-TiO_2_ films show an appreciable amount of surface hole recombination, which is evident from the initial photocurrent overshoot that occurs at the instant light is turned on.

An additional difference between the activities of these films is noted at higher applied potentials of ≥~1 V vs. SCE, which roughly corresponds to 1.23 V vs. RHE in neutral pH (Figure 5b). In this case, particularly for WO_3_-TiO_2_, the electrochemical current rises sharply and is most probably attributable to the onset of electrochemical water splitting/oxygen evolution (OER). A similar effect, although with much lower current densities, is observed for the annealed stainless steel substrate. Thus, the composite films exhibit better electrochemical OER activity. This may simply be an effect of increased surface area [53] or the improved OER mechanistic kinetics of WO_3_ [54].

**Figure 6:**
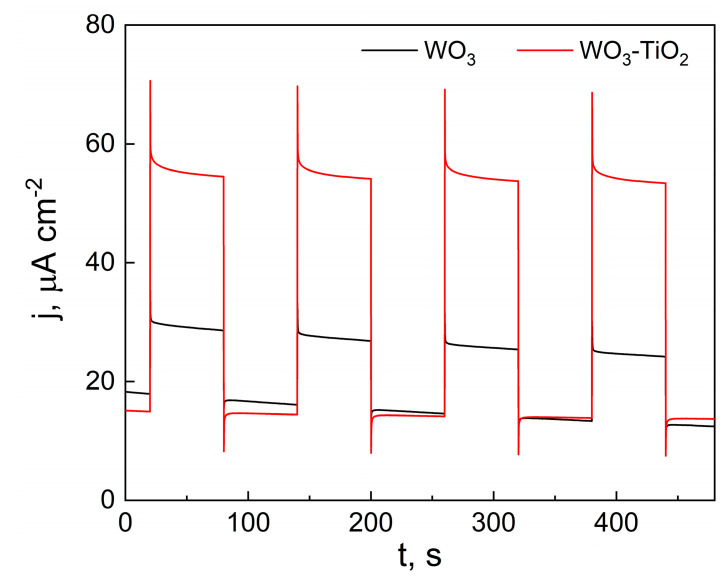
UV on/off pulses in 0.5 M Na_2_SO_4_ of WO_3_ and WO_3_-TiO_2_ films; 365 nm LED, 30 mW cm^−2^ light intensity.

Because the profile of the photocurrent pulse provides useful information about the material photoactivity and surface recombination [55], potentiostatic photocurrent pulses were obtained at 1.2 V vs. SCE and with a longer on/off cycle duration of 120 s (Figure 6). This made it possible to observe the initial photocurrent overshoot immediately upon turning on the light, and the subsequent decay over several pulses.

At the moment the light is turned on, the photocurrent density (j_t=0_) is proportional to the maximum amount of photogenerated electron–hole pairs. For a photoanode, the electrons move to the back contact and are registered as photocurrent, and the holes move toward the electrode–electrolyte interface, where they are either lost to surface recombination or are transferred into the electrolyte to carry out an oxidative photoelectrochemical reaction. After this, the photocurrent decays until a steady state value (j_ss_ is assumed to be the photocurrent at the end of the 60 s pulse) is reached. In this case, it is observed that the WO_3_-TiO_2_ film displays slightly more relative surface recombination than WO_3_. For example, for the WO_3_ film, the j_ss_/j_t=0_ ratio is 0.8; whereas, for the comparable composite film, it is lower at 0.77. However, the WO_3_-TiO_2_ film also generates larger photocurrents overall (~40 µA cm^−2^ compared to just under 12 µA cm^−2^ for WO_3_). A similar result was observed by Castro et al., who found that a hydrothermally synthesized WO_3_ composite with 40 wt% of TiO_2_ exhibited larger photocurrents than WO_3_ [21]. Similarly, WO_3_/TiO_2_ heterostructures on reduced graphene oxide were shown to generate significantly larger photocurrents than the plain films alone [22].

Lastly, similar to the initial jump, there is an “overshoot” to the downside when illumination is turned off. This occurs because of the recombination of holes trapped on the surface with electrons—a phenomenon similar to the decay of the photocurrent.

### 3.3. IMPS Study

IMPS is a non-stationary photoelectrochemical analysis method, modeled by Ponomarev and Peter [56], to determine the features of the space charge region relative to the reaction rate constants. A sinusoidal modulation is applied to the intensity of illumination, and the working electrode under investigation is kept at potentiostatic conditions. In this case, the (photo)current response is proportional entirely to the incident photon flux, i.e., conversion efficiency. It must be noted that here, the light intensity was increased in comparison to previous experiments (30 mW cm^−2^ to 100 mW cm^−2^). This is because the signal for IMPS is not obtained from the entire photocurrent, but from the light-perturbation-induced photocurrent, which corresponds to the photocurrent generated by the amplitude of light intensity modulation (10%). If this light intensity is too low (e.g., 3 mW cm^−2^ as 10% of 30 mW cm^−2^), the spectra can be noisy. However, photogenerated charge carrier recombination is generally larger at higher light intensities [57], so care should be taken to find the optimal measurement conditions for a particular system.

**Figure 7:**
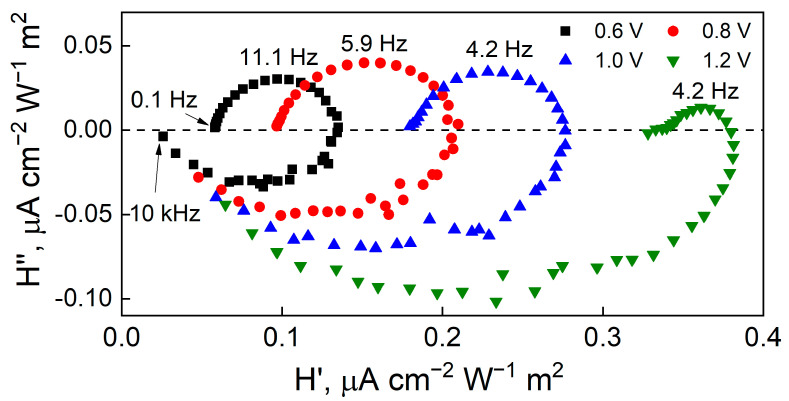
Nyquist plots for IMPS of a WO_3_ film that had been electrodeposited for 5 min, obtained in 0.5 M Na_2_SO_4_, 365 nm LED, 100 mW cm^−2^ light intensity with 10% modulation amplitude.

IMPS Nyquist spectra obtained for WO_3_ are shown in Figure 7, where H represents the complex transfer function of the resulting current measured by controlling the light intensity, normalized to the geometric electrode area. The first quadrant reflects the response from charge-transfer kinetic and surface recombination. The low-frequency intercept H’_LF_ (*left side*) when H’’→0 represents the differential steady-state photocurrent increase due to a differential increase in the light intensity and should be zero if no photoelectrochemical reactions occur. ω_LF_, the radial frequency of the highest point of the recombination semicircle, is a product of first-order rate constants of photogenerated charge transfer and recombination and is inversely related to the recombination time constant.

**Figure 8:**
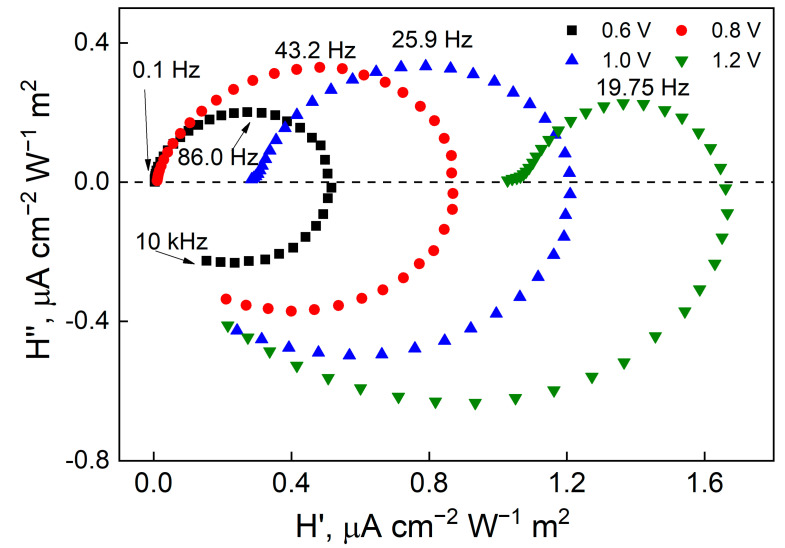
Nyquist plots for IMPS of a WO_3_-TiO_2_ film that had been electrodeposited for 5 min, obtained in 0.5 M Na_2_SO_4_, 365 nm LED, 100 mW cm^−2^ light intensity with 10% modulation amplitude.

Figure 7 for WO_3_ shows a non-zero H’_LF_, suggesting that an electrochemical reaction occurs at all applied potentials. In Figure 8 for WO_3_-TiO_2_ at 0.6 and 0.8 V, this intercept is nearly zero and thus, electron–hole generation and surface recombination primarily occur. A significant shift in this intercept occurs at 1 V where a significant amount of OER is expected to commence driven by the applied potential. The intercept when the imaginary part is zero at a higher frequency (*right side*) represents the amplitude of the flux of charge carriers to the surface. This intercept occurs at a value of H’ that can be expressed as Equation (4).

(4) $$H^{\prime }=j_{h}\frac{C_{H}}{C_{H}+C_{SC}}$$

where *j_h_* is the hole current amplitude, C_H_ and C_SC_ are the Helmholtz and space charge layer capacitances, respectively.

Since the Helmholtz layer size is typically smaller than the width of the depletion, the space charge layer, then it is expected that the Helmholtz capacitance is higher, C_H_ > C_SC_ [58]. Then, this intercept equals j_h_, i.e., the hole current. At higher frequencies relative to this intercept, the influence of the capacitance of the semiconductor space charge dominates and the response moves to the lower quadrant, with a negative imaginary transfer function. The discussed parameters that had been obtained from the spectra are presented in Table 1. The overall trend is that, as the applied potential is raised, H’_LF_ increases because more photogenerated holes are transferred as surface recombination is suppressed. Moreover, the IMPS spectra are not normalized to the amplitude of the photogenerated hole current, and therefore, larger IMPS spectra magnitudes of WO_3_-TiO_2_ suggest higher conversion efficiencies overall, corresponding well to LSV and potentiostatic pulse data.

In IMPS spectra, the high-frequency semicircle occurs because of the RC time constant of the electrochemical cell, i.e., the series resistance of the substrate and the total capacitance. Conversely, the low-frequency semicircle describes the recombination and charge transfer kinetics. Kinetically, these processes are described by first order rate constants: k_tr_—the rate constant of charge transfer across this same interface and k_rec_—the rate of electron–hole recombination at the semiconductor/electrolyte interface. The spectra shown in Figure 5 and Figure 6 exhibit some characteristic tendencies. Most notably—the radius of the low frequency semicircle decreases as the applied potential is increased. In IMPS, the value of the low frequency intercept H’_LF_ corresponds to the DC photocurrent, scaled by the 10% modulation, and is expressed as Equation (5).

(5) $$H_{LF}^{\prime }=j_{h}\frac{k_{tr}}{k_{tr}+k_{rec}}$$

Here, k_tr_/k_tr_ + k_rec_ corresponds to η_trans_—the fraction of hole flux from the space charge region that is involved in the charge transfer reaction, or the transfer efficiency. The transfer efficiency increases with applied potential as photogenerated charge carriers can participate in an electrochemical reaction. Therefore, electron–hole recombination is significant at less positive potentials (the low frequency intercept value is closer to 0), because there is no electrochemical reaction, but at 1.2 V, it decreases considerably with the occurrence of OER.

**Figure 9:**
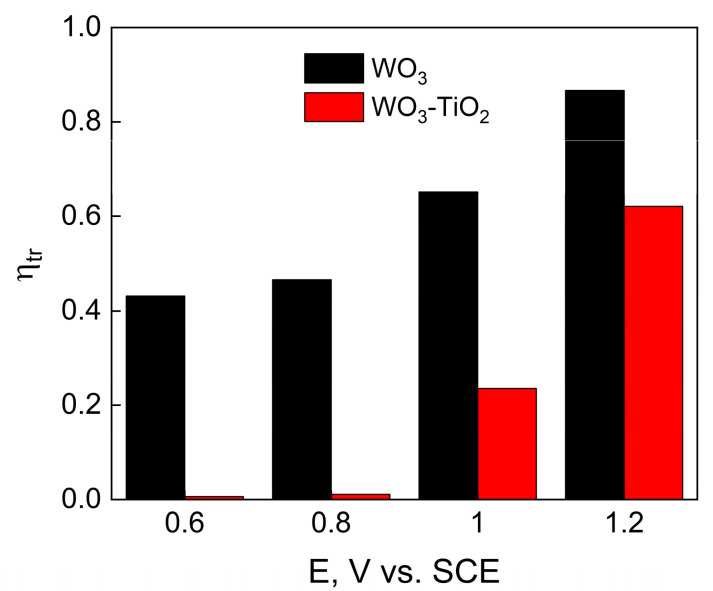
Transfer efficiency values, obtained from IMPS spectra for WO_3_ and WO_3_-TiO_2_ films.

Several important parameters can be calculated from IMPS spectra. Firstly, the transfer efficiency is an indicator of how many of the holes that had arrived at the semiconductor/electrolyte interface that were transferred into the electrolyte to provide the photoelectrochemical reaction. The data for WO_3_ and WO_3_-TiO_2_ films are shown in Figure 9. As expected, the efficiencies are larger with increasing applied potential. Plain WO_3_ films have larger photogenerated transfer efficiency over the entire measured potential range, while the TiO_2_-containing composites have total recombination at lower potentials, but improve rapidly at higher potentials.

To fully discern reaction kinetics, the rate constants should be considered. Both *k_tr_* and *k_rec_* can be calculated from two characteristic points of the IMPS spectrum: the low frequency intercept H’_LF_ (Equation (5)) and ω_LF_, the radial frequency of the highest point of the recombination semicircle, which is described by Equation (6).

(6) $$\omega_{LF}=k_{tr}+k_{rec}$$

**Figure 10:**
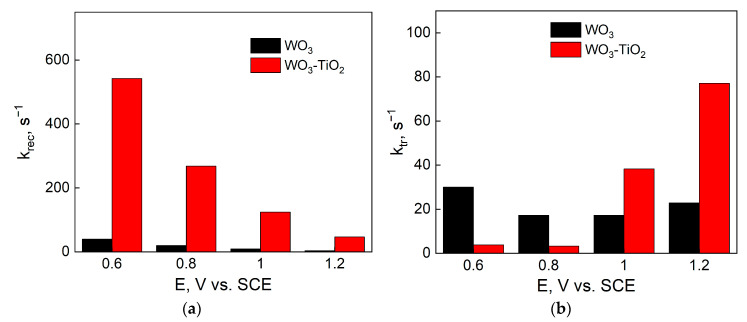
Values of (**a**) k_rec_ and (**b**) k_tr_ for WO_3_ and WO_3_-TiO_2_ films that had been electrodeposited for 5 min.

The spectra were analyzed and the rate constants are presented in Figure 10 in relation to applied potential. Firstly, it can be seen that for both measured photoelectrodes, *k_rec_* decreases with applied potential (Figure 10a), which is expected from the added potential bias as has been discussed earlier.

The k_rec_ values of WO_3_-TiO_2_ are larger, showing faster recombination kinetics. However, when considering the kinetic transfer constant, it is apparent that *k_tr_* increases significantly for the composite films at higher potentials, compared to WO_3_ (Figure 10b). Thus, there is a higher OER reaction rate on the WO_3_-TiO_2_ under a biased photoelectrochemical environment. At an applied potential of 1.2 V vs. SCE, the k_tr_ of the composite is three times larger than that of the respective WO_3_ film, consistent with the voltammetry result in Figure 5 and the chronoamperometry in Figure 6.

While the addition of discrete TiO_2_ nanoparticles to WO_3_ generated a higher photocurrent compared to WO_3_ alone, it had the negative effect of increasing the recombination rate constant, particularly near open circuit conditions at small applied potentials. The increased recombination rate may be due to the introduction of grain boundaries and local potential perturbation by the particles. The recombination rate constant decreased with applied potential, and in the applied potential range for conventional electrolysis, the WO_3_-TiO_2_ composite greatly exceeded the performance of the WO_3_. At an even larger applied potential exceeding 1.2 V vs. SCE, evident in the polarization curve in Figure 5, the WO_3_-TiO_2_ composite OER current density greatly exceeded WO_3_ even without light; thus, the TiO_2_ addition to WO_3_ improved its electrocatalytic performance. Georgieva et al. [30] also found an enhanced photocatalysis for TiO_2_/WO_3_ layers on stainless steel compared to WO_3_ but attributed the enhancement to reduced recombination. The results presented here are in contrast to this reasoning, elucidated by the additional evidence from IMPS. The enhancement in our work is due to larger charge transfer kinetics and not suppressed recombination.

## 4. Conclusions

In this study, WO_3_ and composite WO_3_-TiO_2_ films were prepared by electrodeposition from an acidic peroxo-tungstate precursor solution containing suspended anatase TiO_2_ nanoparticles for the formation of the composite. The annealed films were found to be crystalline, with WO_3_-TiO_2_ having a distinct monoclinic WO_3_ structure, but plain WO_3_ displaying characteristics of hydrate formation. XPS showed the dominance of W^6+^ in both samples. In addition, the inclusion of Ti^4+^ and a small amount of Ti^3+^ was proven by deconvoluting the Ti 2p core level spectra. The films prepared in this study were found to be photoelectrochemically active, and WO_3_-TiO_2_ generated significantly larger photocurrent magnitudes compared to the WO_3_ alone. However, the WO_3_-TiO_2_ composite photocurrents possessed considerable recombination at and near open circuit conditions. While the overall transfer efficiency is higher for WO_3_, applying a bias potential resulted in a significant increase in the OER current density of the WO_3_-TiO_2_ composite: it is three times higher at an applied potential of 1.2 V vs. SCE, and an associated expected decrease in the electron–hole recombination rate. The implications of these results show that electrodeposition of a WO_3_-TiO_2_ composite from an electrolyte containing a peroxo-tungstate precursor and TiO_2_ nanoparticles result in significant changes in the material’s structure, morphology, and consequently, PEC activity. The synthesis of composites is facile, they exhibit improved PEC performance as photoanodes when compared to WO_3_, but also suffer from higher photogenerated electron–hole recombination at lower applied potentials.

## Figures and Tables

**Table 1:** Parameters, obtained from interpretation of IMPS Nyquist plots.

	WO_3_			WO_3_-TiO_2_		
E, V	ω_LF_, Rad s^−1^	H’_LF_, µA cm^−2^ W^−1^ m^2^	j_h_, µA cm^−2^ W^−1^ m^2^	ω_LF_, Rad s^−1^	H’_LF_, µA cm^−2^ W^−1^ m^2^	j_h_, µA cm^−2^ W^−1^ m^2^
0.6	69.6	0.058	0.135	546.2	0.004	0.511
0.8	37.0	0.097	0.208	271.3	0.010	0.867
1.0	26.4	0.180	0.276	162.7	0.284	1.208
1.2	26.4	0.330	0.378	124.0	1.028	1.653

## Data Availability

Dataset available on request from the authors.
